# Synthesis of nano-fibers containing nano-curcumin in zein corn protein and its physicochemical and biological characteristics

**DOI:** 10.1038/s41598-020-73678-w

**Published:** 2021-01-21

**Authors:** Narges Fereydouni, Jebrail Movaffagh, Nafise Amiri, Susan Darroudi, Aida Gholoobi, Arash Goodarzi, Alireza Hashemzadeh, Majid Darroudi

**Affiliations:** 1 Department of Tissue Engineering, School of Medicine, Fasa University of Medical Sciences, Fasa, Iran; 2 Noncommunicable Diseases Research Center, Fasa University of Medical Sciences, Fasa, Iran; 3 Department of Medical Biotechnology and Nanotechnology, School of Medicine, Mashhad University of Medical Sciences, Mashhad, Iran; 4 Department of Pharmaceutical Nanotechnology, School of Pharmacy, Mashhad University of Medical Sciences, Mashhad, Iran; 5 Targeted Drug Delivery Research Center, Pharmaceutical Technology Institute, Mashhad University of Medical Sciences, Mashhad, Iran; 6 Student Research Committee, International UNESCO Center for Health-Related Basic Sciences and Human Nutrition, Mashhad University of Medical Sciences, Mashhad, Iran; 7 Medical Genetics Research Center, School of Medicine, Mashhad University of Medical Sciences, Mashhad, Iran; 8 Department of Medical Physiology, Faculty of Medicine, Mashhad University of Medical Sciences, Mashhad, Iran; 9 Metabolic Syndrome Research Center, Mashhad University of Medical Sciences, Mashhad, Iran; 10 Nuclear Medicine Research Center, Mashhad University of Medical Sciences, Mashhad, Iran

**Keywords:** Biotechnology, Cell biology, Chemical biology, Medical research

## Abstract

Curcumin contains many biological activities as a natural bioactive substance, however, its low solubility stands as a huge bioavailability disadvantage. Recently, different methods have been developed for utilizing the tremendous medicinal properties of this material. In this study, an Oil/Water nano-emulsion of curcumin (Nano-CUR) has been woven in zein polymer at three percentages of 5%, 10%, and 15% (v/v). We have investigated the physicochemical properties of nanofibers (NFs) including FESEM, FTIR, tensile strength, encapsulation efficiency, and release profile, as well as biological properties. According to the data, the NFs have been observed to become significantly thinner and more uniformed as the involved percentage of Nano-CUR had been increased from 5 to 15%. It is considerable that the tensile strength can be increased by heightening the existing Nano-CUR from 5% towards 15%. The resultant NFs of zein/Nano-CUR 15% have exhibited higher in vitro release and lower encapsulation efficiency than the other evaluated zein/Nano-CUR NFs. It has been confirmed through the performed viability and antioxidant studies that zein/Nano-CUR 10% NFs are capable of providing the best conditions for cell proliferation. Considering the mentioned facts, this work has suggested that Nano-CUR can be successfully woven in zein NFs and maintain their biological properties.

## Introduction

As a branch of nanomaterials, nano-fibers (NFs) have been widely used for biomedical and many other applications throughout the recent years due to their intrinsic properties^1^. Relatively, NFs are classified as carriers with a nanostructure diameter of less than 100 nm, while the fibers that contain a diameter of less than 1000 nm are also included in this category; the measurement of these materials is commonly done by the employment of electrospinning technique. However, a larger surface can be constructed by reducing the diameter of fibers from micrometer to under micrometer or nm^2^.

NFs can be synthesized from natural and synthetic polymers or their different compositions. In addition, they can be efficiently delivered by both hydrophilic and hydrophobic drugs^3,4^. Different parameters can modulate the process of NFs drug release such as the ratio of drug to polymer, diameter, morphology, and/or NFs porosity^2^. Among the available methods for preparing NFs^5–7^, the electrospinning technique stands as the most accepted one since it has proved to be cost-effective and contain a high efficiency for producing these materials in the range of nm to micro-meters with varying molecules^8^.

Due to the encapsulation of drug molecules in a polymer structure, NFs can function as a drug delivery system, be applicable for skin treatments, and contribute to simple releases that take place throughout topical and systemic procedures^9^. In order to provide a proper wound healing, several parameters are required to be considered such as air conditioning, preventing the entrance of microorganisms into wound bed, proper dressing, and adhesion to the wound site^10^. Hereof, NF-based systems can benefit this process in several ways such as facilitating better conditions for the cellular respiration through their high permeability and provide an ideal environment for the wounds to be repaired. The moisture that exists under wound dressing can help the healing procedure and supply a suitable barrier for the ulcerative exudates, which are responsible for enclosing proteins and protective cytokines in the wound. The rate of vapor transmission through the wound stands as another significant parameter for choosing the proper materials^11–13^.

Plant proteins are known to be low immunogenic, low-cost, affordable, and highly biocompatible, which are commonly extracted from plants or the byproducts of agricultural industry^14^. Some of these proteins, such as soy protein, zein (corn), and gluten (wheat), are being currently used in food packaging industry, while their potential for being applied in health applications is being examined as well. Being a corn protein, zine is a byproduct of bioethanol industry that has been widely studied in regards to the biomedical applications^15^. This particular material contains certain valuable properties such as hardness, flexibility, hydrophobicity, anti-bacterial resistance, and anti-oxidant activity^15–17^. Miyoshi et al*.*, have initially employed the technique of electrospinning for the purpose of synthesizing NFs and thereafter, investigated the existing relationship between the inverse ratio of polymer concentration and electric potential energy, as well as their effects on the properties of NFs. The concentration of zein solution that had been applied in the synthesis of NF has been evaluated at 18–60%^18^. It has been indicated by the work of Torres-Giner et al*.* that the acidic solution of zein can produce smoother NFs, while the alkaline solution leads to the formation of mold-fiber beaded structures^19^.

By the usage of NFs based systems, various natural and synthetic materials have been investigated for wound healing throughout the recent years. Curcumin is a natural polyphenolic material that is extracted from the root of *Curcuma longa* (Zingiberaceae family) and has been widely utilized as a medicinal plant in many Asian countries over the past years^20^. It is applied in traditional medicine for the treatment of different diseases such as anorexia, cough, and biliary problems, as well as liver and sinus illnesses^21^. Many studies have reported the antibacterial, anti-proliferation, anti-inflammatory, anti-oxidant, anti-cancer, and anti- amylogenic properties of curcumin in cellular and animal models^22–24^. Other activities that have been assigned to curcumin include anti-rheumatic, anti-viral, liver, anti-HIV, and wound healing features^25^. Furthermore, curcumin and tumeric have been approved as safe products by the FDA, FAO, and WHO organizations^26^. There has not been any reported side effects from the oral administration of this material in doses that reach up to 12 g per day throughout the clinical trials^27^.

Curcumin is a hydrophobic polyphenol that contains certain properties in low degrees, such as low solubility in water, low stability, rapid metabolism, low absorption, and availability, which have reduced its medical benefits in great extents^28^. To overcome these obstacles, drug delivery systems have been considered since they can prolong the flow time, permeability, and resistance towards metabolic degradation^29–31^. One of these methods would be the determination of synthesized curcumin in a scaffold bed of NFs, while up to the present, this material has been woven in the NFs mats that are composed of different polymers^32–43^. Researchers have prepared various wound dressing nanocomposites that had been consisted of curcumin-NFs in order to understand the mechanism of repairing diabetic ulcers, burns, and etc. by curcumin-loaded NFs, examine the physicochemical properties and release of curcumin from the polymer bed, and investigate the process of wound healing. It has been suggested by the obtained results that the process of wound healing with curcumin-NFs may occur by preserving the antioxidant, anti-inflammatory, and anti-bacterial properties of curcumin, as well as the inherent properties of polymer scaffolds^44^. However, there are still limitations to the solubility and stability of curcumin although many methods such as liposome^45^, polymeric nanoparticles^46^, solid lipid nanoparticles (SLNs)^47^, albumin nanoparticles^48^, microemulsions and nanoemulsions^49^, nanospheres, and microcapsules^50^ have been devised in recent years to obtain a higher aqueous solubility, bioavailability, activity, and lower toxicity^28,51^. In this regard, the emulsion method has received much attention in recent decades due to containing a simple synthesizing process and high efficiency, as well as requiring low costs, and eco-friendly materials^52–56^. This method has been exerted to synthesize water-insoluble substances^57,58^, oils^59,60^, plant extracts^61,62^, proteins^63^, nucleic acids^64^, curcumin, and many other substances^49^ for drug delivery intentions, while some of these materials have been evaluated even in nanofiber scaffolds^65–67^. According to the available data, there has not been any studies conducted on the usage of O/W nano-emulsion of curcumin in nanofiber substrates. Therefore, we have attempted in this work to investigate the possibility of O/W nano-emulsion curcumin (Nano-CUR) loading in zein nanofibers and evaluate the physicochemical properties, release, and cytotoxicity of zein/Nano-CUR NFs.

## Results

### Morphological study

**Figure 1:**
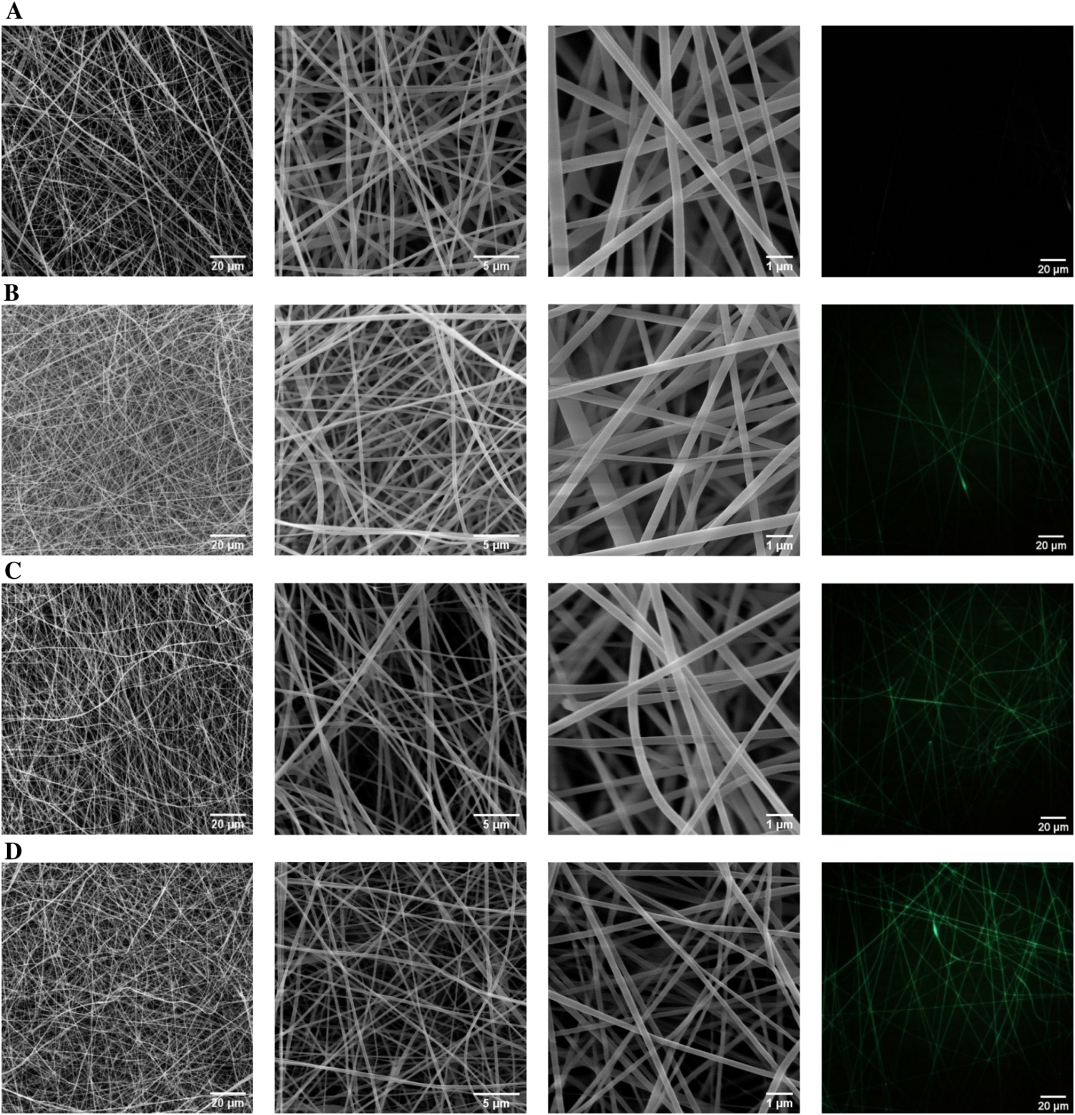
FESEM images of (**A**) zein NFs, (**B**) zein/Nano-CUR 5% NFs, (**C**) zein/Nano-CUR 10% NFs, (**D**) zein/Nano-CUR 15% NFs.

The morphology of electrospun NFs has been the first feature of these membrane models that had been aimed to be investigated. In this study, the Nano-CUR, which had been successfully prepared in our previous work, has been loaded in zein polymer through the means of electrospinning method while applying the appropriate parameters including solvent, the concentrations of final polymer and Nano-CUR, and flow speed. Figure 1 demonstrates the FESEM micrographs of zein NFs that had contained the varying Nano-CUR volumes of 5%, 10%, and 15% (v/v). According to our observations, the existing NFs have become significantly thinner and more uniformed as the percentage of Nano-CUR had been increased from 5 to 15%. It has been also indicated that Nano-CUR had been thoroughly mixed with the polymer solutions due to the lack of detecting any Nano-CUR aggregates throughout the surface of NF.

**Figure 2:**
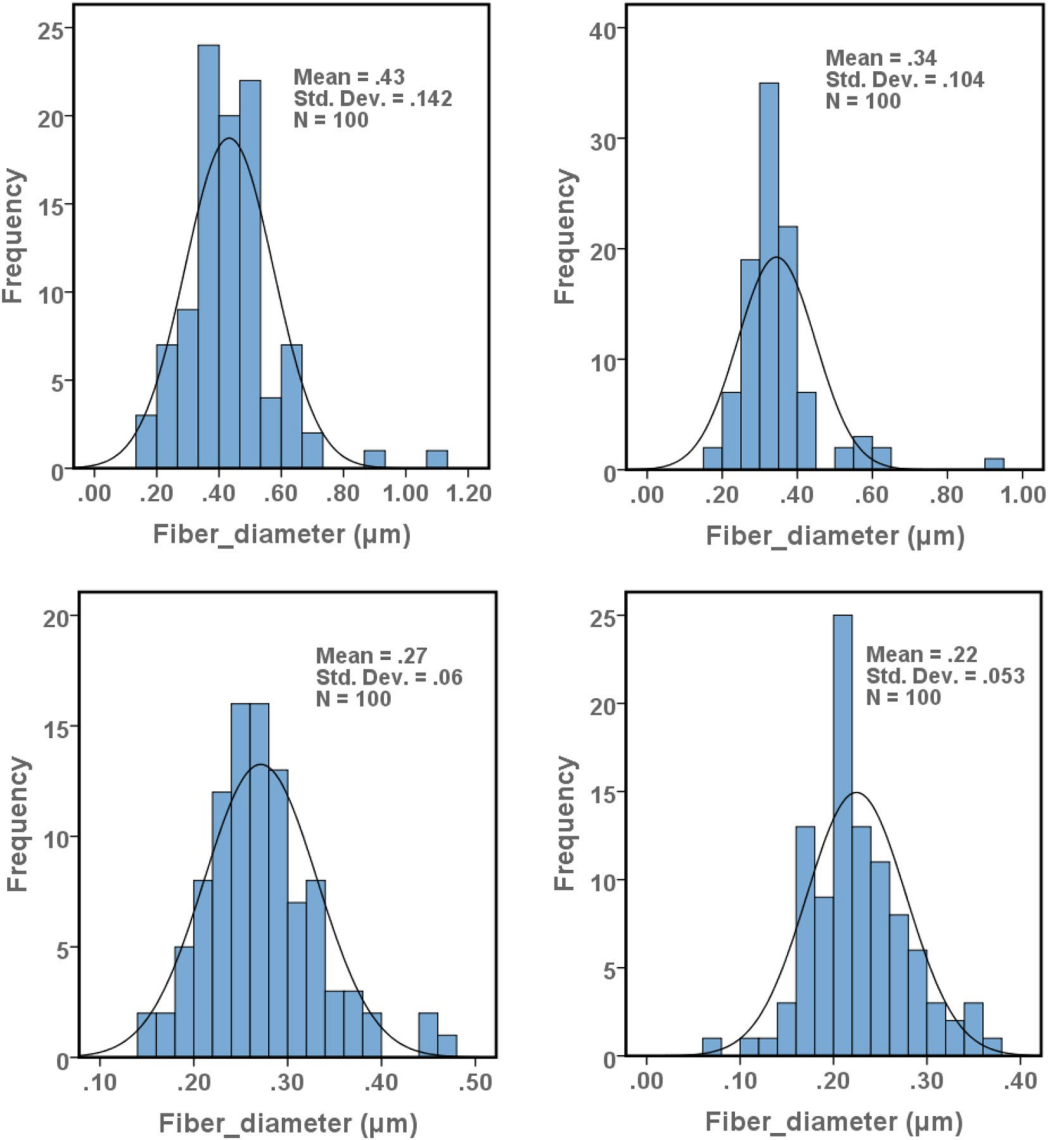
Size distribution of (**A**) zein NFs, (**B**) zein/Nano-CUR 5% NFs, (**C**) zein/Nano-CUR 10% NFs, (**D**) zein/Nano-CUR 15% NFs.

As it is displayed in Fig. 2 and Table 1, the mean diameter and diameter distribution of NFs have faced a significant reduction upon the addition of Nano-CUR to the zein solution. However, it has been perceived in the obtained outcomes that the electrospinning of zein/Nano-CUR solution in a similar volume has resulted in different thicknesses of NFs. Furthermore, the thicknesses and porosity percentages of zein/Nano-CUR 10% (*p* < 0.05) and 15% NFs (*p* < 0.001) have been significantly higher than that of the zein blank NFs (*p* < 0.01). In addition, the viscosity of as-prepared zein/Nano-CUR 5%, 10%, and 15% solutions has faced a notable decrease when compared to the zein solution (*p* < 0.001).

**Table 1:** Physicochemical properties of zein and zein/Nano-CUR 5%, 10%, and 15% NFs.

	Zein NF	Zein/Nano-CUR 5% NF	Zein/Nano-CUR 10% NF	Zein/Nano-CUR 15% NF	Open container
Diameter (nm)	430 ± 142	340 ± 104^a^	270 ± 60^ab^	220 ± 53^abc^	
Thickness (mm)	0.082 ± 0.01	0.0964 ± 0.01	0.112 ± 0.01^a^	0.133 ± 0.02^ab^	
Viscosity (Pa.s)	0.534 ± 0.03	0.373 ± 0.04^a^	0.274 ± 0.02^ab^	0.267 ± 0.02^ab^	
Porosity (%)	28.3 ± 6.5	33.2 ± 4.5	34.6 ± 6.4^a^	35.2 ± 2.4^a^	
WVTR (g/m^2^)	1680.64 ± 248.34	1840.00 ± 290.35	2005.26 ± 250.36	2550.27 ± 246.25^ab^	5355.25 ± 357.28
Water-uptake capacity	29.87 ± 1.57	32.23 ± 2.12	35.64 ± 3.14^a^	37.34 ± 2.15^ab^	

Data displayed as mean ± SD. a: significant difference between zein/Nano-CUR 5%,10%, and 15% NFs in comparison with zein NF, b: significant difference between zein/Nano-CUR 10%, and 15% NFs in comparison with zein/Nano-CUR 5% NF, c: significant difference between zein/Nano-CUR 15% NFs in comparison with zein/Nano-CUR 10% NF.

We have observed the fluorescence of nanofibers through the application of a fluorescent microscope (DP73) (Fig. 1), while the fluorescent emission of zein nanofibers had been collected within the range of green light wavelength. Zein NFs and zein/Nano-CUR NFs have exhibited green fluorescent signals without using any external fluorescent dyes, however, the intensity of fluorescent has been significantly increased as the volume of Nano-CUR had been enlarged. Next to discovering the even distribution of Nano-CUR throughout the nanofibers, we have indicated their lack of accumulation by observing the uniformed fluorescent signals. These results are suggestive of Nano-CUR high compatibility with zein polymer and the formation of a homogeneous solution even in the course of spinning step.

### FTIR study

**Figure 3:**
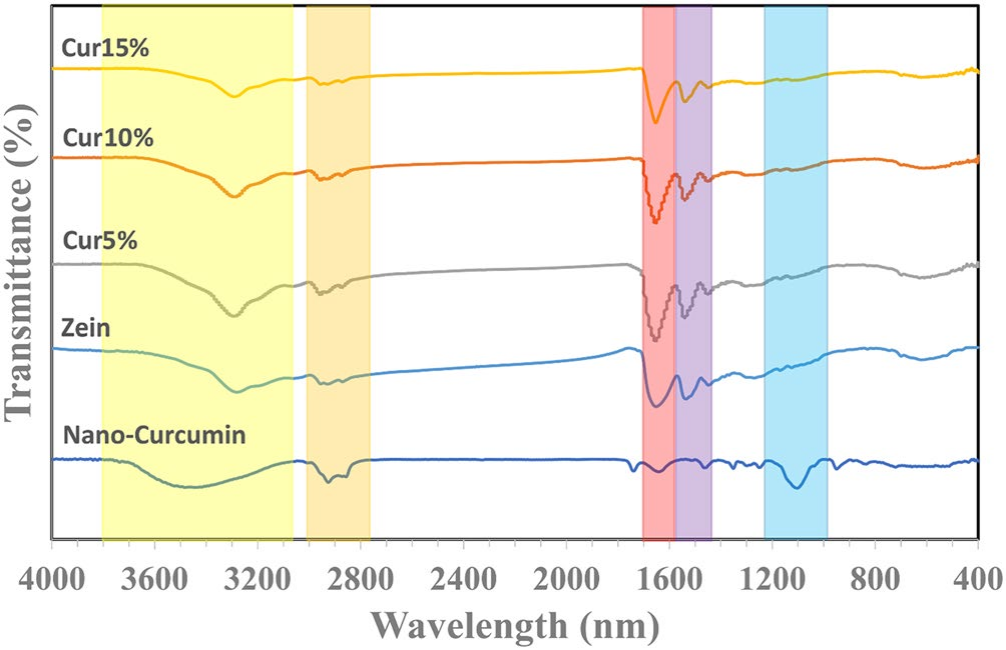
FTIR image of Nano-CUR, zein NFs, zein/Nano-CUR 5% NFs, zein/Nano-CUR 10% NFs, zein/Nano-CUR 15% NFs.

Figure 3 illustrates the FTIR spectra of Nano-CUR, zein, and zein/Nano-CUR 5%, 10%, and 15% NFs, which had been obtained to determine the inter-molecular interactions and composition. The structure of zein NFs has been characterized by the utilization of amide vibrations at 1650 cm^−1^ (amide I) and 1536 cm^−1^ (amide II)^68^. The available spectrum of Nano-CUR, which had been previously pointed out at 3484 cm^-1^, 2925 cm^-1^, and 1103 cm^-1^, has been related to the existing vibration stretching of O–H, C-H, and C-O groups^49,69^. In regards to the case of zein/Nano-CUR NFs, we have detected the appearances of typical amide I and II bands at around 1650 and 1536 cm^−1^, respectively.

### Mechanical measurement

**Figure 4:**
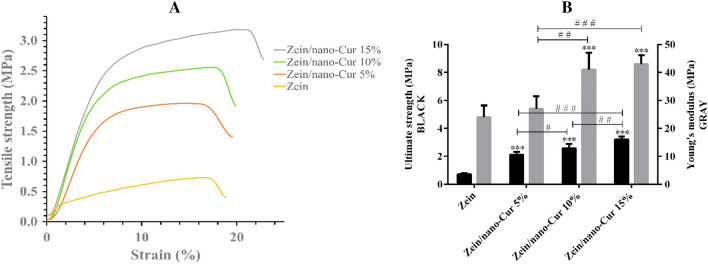
The stress–strain diagram (**A**) and the ultimate strength and young’s modulus parameters (**B**) of zein and zein/Nano-CUR 5%, 10%, and 15% NFs; # indicates significance between groups and * indicates significance of every group with zein group (^#/*^p < 0.05, ^# #/**^p < 0.01, and ^# # #/***^p < 0.001).

We have assessed the mechanical properties of zein and zein/Nano-CUR NFs, which had included Young's modulus, elongation percentage at break point, and tensile strength. Figure 4A depicts the stress–strain diagrams of zein and zein/Nano-CUR NF scaffolds. The ultimate tensile strength of zein and zein/Nano-CUR 5%, 10%, and 15% NFs have been 0.73 ± 0.06, 2.11 ± 0.2, 2.58 ± 0.3, and 3.21 ± 0.21 MPa, respectively. According to the results, the ultimate tensile strength of zein NFs has been significantly heightened as the amount of Nano-CUR had been increased (*p* < 0.001) (Fig. 4B). The young's modulus of zein and zein/Nano-CUR 5%, 10%, and 15% NFs have been measured to be 24 ± 4.2, 27 ± 4.5, 41 ± 6.1, and 43 ± 3.2 MPa, respectively. The Young's modulus of zein/Nano-CUR 10% and 15% has been significantly increased in comparison to the zein NFs (*p* < 0.001) (Fig. 4B). Moreover, the elongation (%) at break point have been detected to be 17.06% ± 5.3%, 11.36% ± 2.2%, 17.29% ± 3.1%, and 20.51% ± 5.1%, respectively, which had been indicative of the higher percentage of zein/Nano-CUR 15% NF in comparison to that of the zein/Nano-CUR 5% NF (*p* < 0.05). The obtained outcomes from the performed mechanical examination have suggested that increasing the portion of Nano-CUR can result in extending the mechanical properties of NFs.

### Drug EE% and release assessment

**Figure 5:**
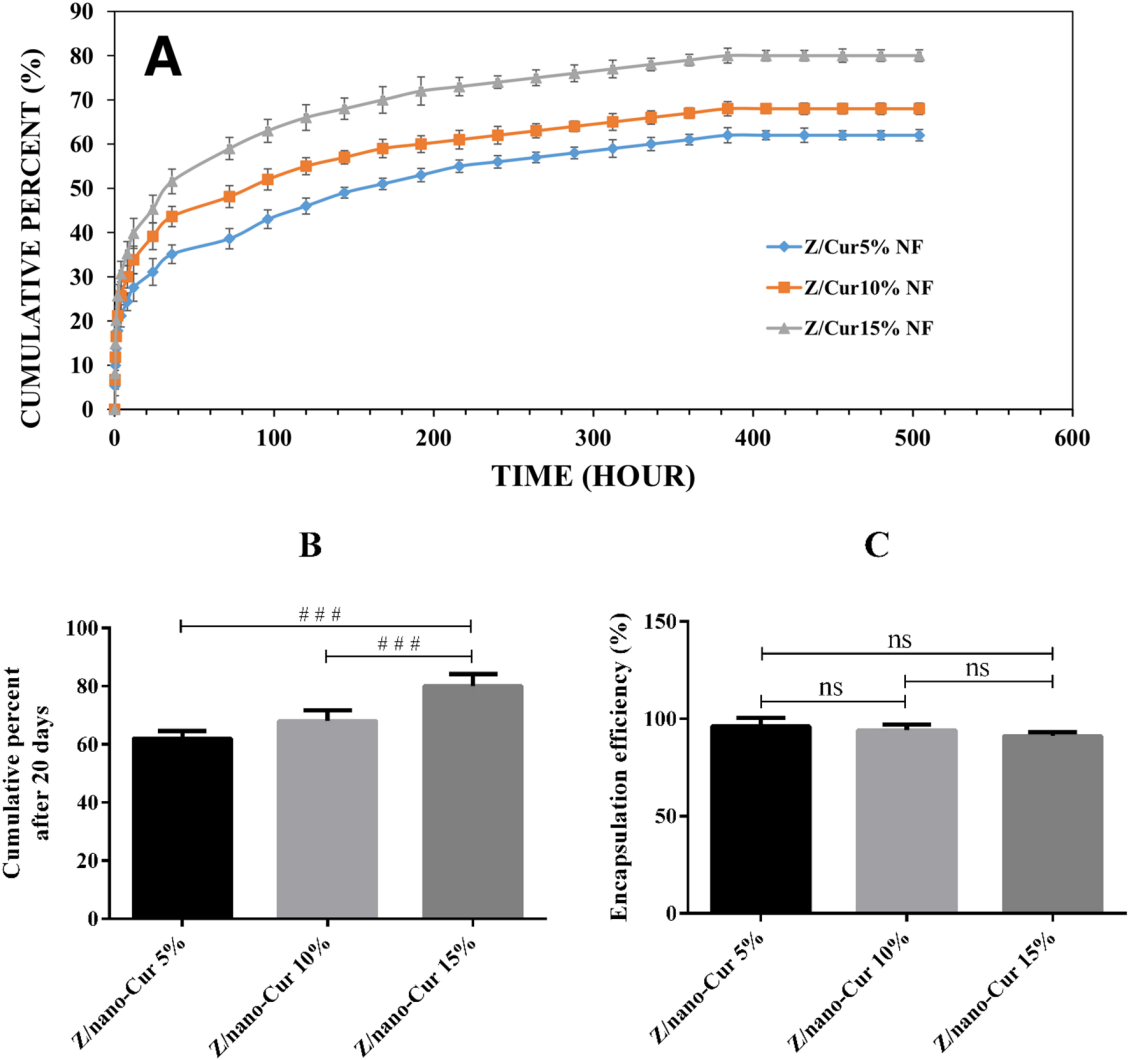
The release diagram of Nano-CUR 5%, 10%, and 15% from zein NFs in 20 days (**A**), in vitro release percentage (**B**), and encapsulation efficiency diagrams (**C**) of zein/Nano-CUR 5%, 10%, and 15% NFs; # indicates significance between groups and * indicates significance of every group with zein group (^#/*^p < 0.05, ^# #/**^p < 0.01, and ^# # #/***^p < 0.001).

The release profile of Nano-CUR that had been obtained from electrospun zein/Nano-CUR 5%, 10%, and 15% NFs is demonstrated in Fig. 5A. Although the release models in regards to all of the involved zein/Nano-CUR 5%, 10%, and 15% NFs have been similar, yet the electrospun zein/Nano-CUR 15% NFs had displayed a higher release rate than the zein/Nano-CUR 5% and 10% NFs. Zein/Nano-CUR 5%, 10%, and 15% have released 62% ± 2.6%, 68% ± 3.7%, and 80% ± 4.2% of Nano-CUR in 20 days, respectively. The observed significant difference between zein/Nano-CUR 15% NFs, 5% NFs (*p* < 0.001), and 10% NFs (*p* < 0.001) is exhibited in Fig. 5B. Zein/Nano-CUR 5%, 10%, and 15% NFs has displayed a burst release of 40%, 34%, and 28% at the first time interval of 12 h, respectively, which had been continuous until the release became sustained and reached a plateau phase. According to Fig. 5C, the EE% of zein/Nano-CUR 5%, 10%, and 15% NFs have been 96% ± 4.42%, 94% ± 3.04% and 91% ± 2.01%, respectively, without displaying any significant differences.

### WVTR

The amount of water vapor transmission (WVTR) in g/m^2^ regarding zein and zein/Nano-CUR nanofiber scaffolds of 5%, 10%, and 15% are illustrated in Table 1. The WVTR of zein and zein/Nano-CUR 5%, 10%, and 15% have been 3220.64 ± 248.34 g/m^2^, 2550.27 ± 246.25 g/m^2^, 2005.26 ± 250.36 g/m^2^, and 1840.00 ± 290.35 g/m^2^, respectively. The performed statistical studies have indicated that the WVTR of zein NFs had been significantly higher than the scaffold of zein/Nano-CUR 10% and 15%.

### Water-uptake capacity

The amount of water-uptake capacity percentages of scaffolds in regards to zein and zein/Nano-CUR nanofiber scaffolds of 5%, 10%, and 15% are presented in Table 1. The water-uptake capacity percentages of zein and zein/Nano-CUR 5%, 10%, and 15% have been measured to be 29.87 ± 1.57%, 32.23 ± 2.12%, 35.64 ± 3.14%, and 37.34 ± 2.15%, respectively. According to the statistical analysis, the water-uptakes of zein/Nano-CUR 10% and 15% have been significantly higher than that of the zein NFs.

### Cytotoxicity test

**Figure 6:**
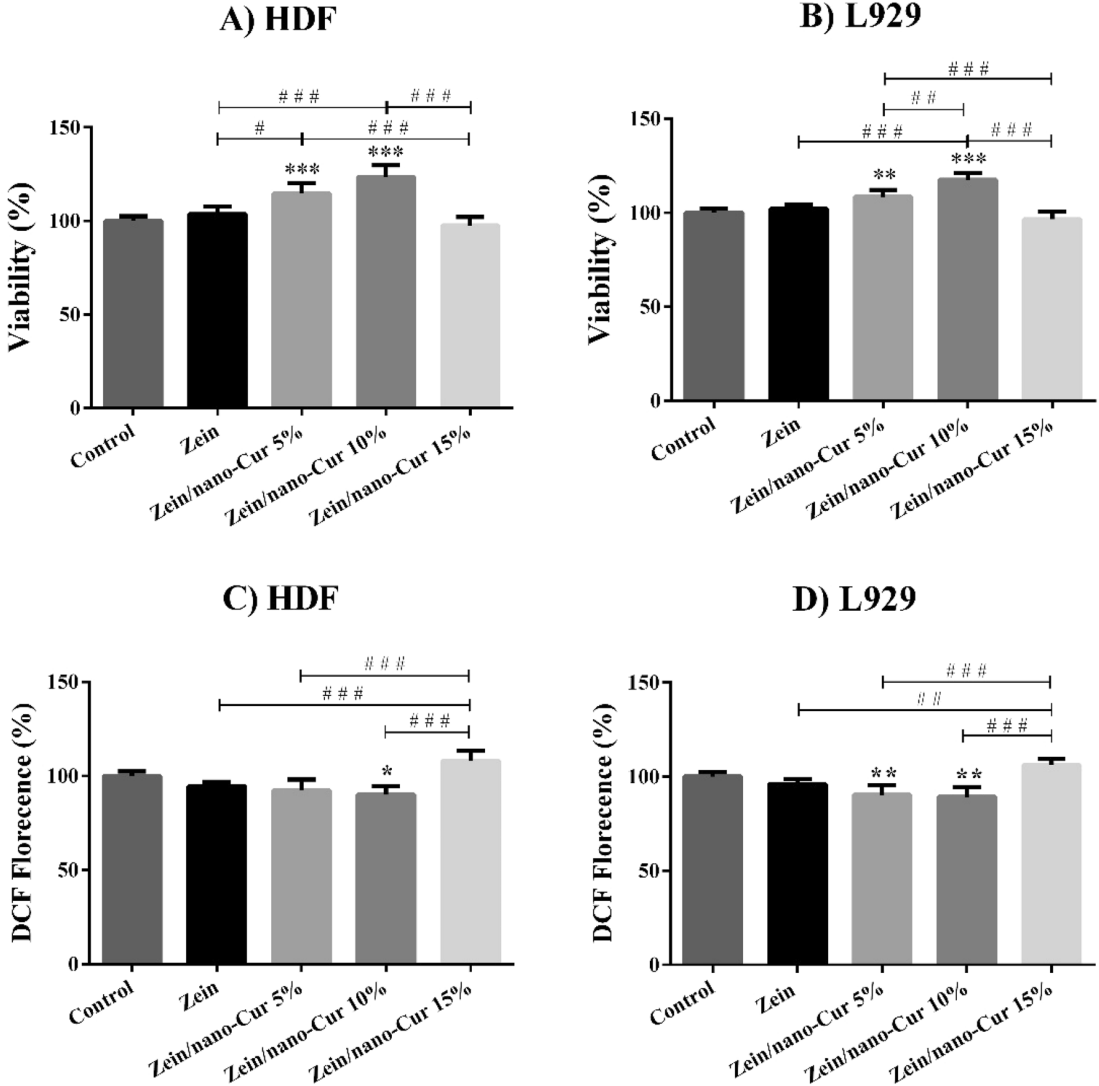
Viability (**A, B**), and oxidative stress (**C, D**) diagram of zein and zein/Nano-CUR 5%, 10%, and 15% NFs on HDF and L929 cell lines; # indicates significance between groups and * indicates significance of every group with zein group (^#/*^p < 0.05, ^# #/**^p < 0.01, and ^# # #/***^p < 0.001).

We have evaluated the process of cell proliferation on zein and zein/Nano-CUR 5%, 10%, and 15% NFs through the usage of colorimetric MTT assay (Fig. 6A-B). In comparison to the case of control group, zein/Nano-CUR 5% and 10% mats have exhibited a significant increase in proliferation throughout the L929 and HDF cell lines.

### Intracellular ROS

The intracellular free radicals of zein and zein/Nano-CUR 5%, 10%, and 15% NFs have been assessed by the application of DCFH-DA colorimetric assay (Fig. 6C-D) and according to the gathered results, zein/Nano-CUR 10% has been capable of significantly reducing the existing free radicals in HDF cell media when compared to the control group. Also in comparison to the control group, the number of free radicals in L929 cell media has been significantly reduced by zein/Nano-CUR 5% and 10%.

### Antibacterial study

**Figure 7:**
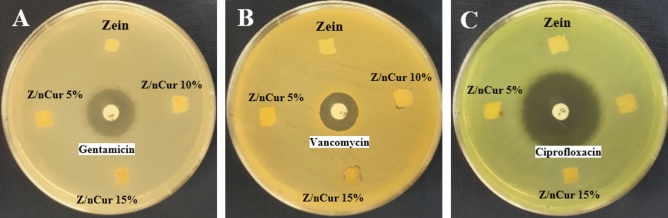
Evaluation of antibacterial properties of zein and zein/Nano-CUR NFs. (**A**) *Escherichia coli*, (**B**) *Staphylococcus aureus*, (**C**) *Pseudomonas aeruginosa.*

The antibacterial properties of zein and zein/Nano-CUR NFs have been determined by the means of agar diffusion method (Fig. 7A-C). The antibiotic discs vancomycin, ciprofloxacin, and gentamicin have exhibited a 16 mm, 30 mm, and 20 mm growth of inhibition zones, respectively, whereas zein and zein/Nano-CUR NFs had not displayed any growth of inhibition zone on *Escherichia coli*, *Pseudomonas aeruginosa*, and *Staphylococcus aureus*.

## Discussion

There are several studies about the fabrication of curcumin in different polymers through the employment of electrospinning techniques and relatively the aim of this study has been set to demonstrate the possibility of developing the oil-in-water (O/W) nano-emulsion of curcumin (Nano-CUR), which would be loaded with zein NFs, and also evaluate their in vitro activity. Although curcumin is a hydrophobic polyphenol molecule with remarkable biological properties, yet due to containing a very low water solubility, many studies have attempted to increase its solubility and control its release for exerting its unique features in medical applications^70,71^. In this work, we have utilized the O/W nano-emulsion of curcumin (Nano-CUR) while assuming that this alteration would be able to increase the chemical stability and solubility of curcumin and turn the resultant into a more effective substance in biological environment due to the small size and extended ratio of surface to volume.

The first aspect of NF studies has been performing investigations on the effects of electrospinning parameters on the NF morphology and diameter. According to our observations, NFs become significantly thinner and more uniformed as the portion of Nano-CUR is increased from 5 to 15%. The obtained results has also indicated that the volume of porosity increases as the amount of Nano-CUR is enlarged, which could have been caused by the induced reduction in the diameter of nanofibers, as well as the improved uniformity of fibers and increased thickness of nanofibers. Subsequent to arranging similar conditions and evaluating the effective factors, the reduction of viscosity and extending spinnability have been the only parameters that had been observed to be capable of reducing the diameter and increasing the thickness. In comparison to the oily phase, oil/water emulsion Nano-CUR can reduce the viscosity of final solution through the addition of five times more water. The gathered outcomes have indicated that the viscosity of final zein solution has been reduced from 0.534 Pa.s to 0.267 Pa.s in the final zein/Nano-CUR 15% solution, suggesting that the viscosity of all the involved samples had been within the range of producing bead-free nanofibers. Meanwhile, our pilot attempt to produce zein/Nano-CUR 20% has ​​led to the production of high beaded nanofibers, which is not mentioned in the results of this study. To the extent of our knowledge, surface tension is the predominant factor in a very low viscosity situation, while the spraying of polymer solution can lead to the formation of beaded nanofibers^72^. Furthermore, numerous studies have reported that increasing and reducing viscosity results in expanding and decreasing the diameter of nanofibers, respectively^1,73^. Having a higher evaporation rate of acetic acid than water could be considered as another reason behind the occurrence of an increase in the diameter of nanofibers as the volume of Nano-CUR is reduced, since this alteration can cause an increase in the viscosity of polymer solution at the tip of the needle and during the traveling of nanofibers towards being deposited on the collector^74^.

Next to the even distribution of Nano-CUR throughout the nanofibers, we have confirmed their lack of accumulation by observing the uniform fluorescent signals. Various studies have reported the fluorescent properties of curcumin^75,76^, which can be also detected by the usage of UV illumination or confocal laser scanning microscope (CLSM)^68^. Furthermore, our study has displayed the capability of oil/water Nano-CUR in exhibiting the fluorescent signals in zein nanofibers, which is indicative of their high compatibility with zein polymer, the formation of a homogeneous solution even in the course of spinning, and confirming the stability of zein/Nano-CUR homogenous solution during the electrospinning process.

There has not been any notable differences between the zein and zein/Nano-CUR spectra in the FTIR spectra, which may be due to the compatibly entrapment of Nano-CUR into zein NFs. The lack of observing any new peaks in the zein/Nano-CUR NFs has indicated the existence of a simple combination that does not contain any chemical bonds.

We have evaluated the factors of Young's modulus, elongation percentage at break point, and tensile strength for assessing the mechanical parameters of zein and zein/Nano-CUR NFs and according to the obtained outcomes, they can be improved by increasing the amount of Nano-CUR. As it has been previously stated, the diameter of NFs faces a reduction through the addition of Nano-CUR to zein scaffold; therefore, it seems to be quite logical to assume that causing a reduction in the average diameter of NFs and extending the number of nanofibers per unit area from zein/Nano-CUR 5% to 15% would increase the tensile strength and young's modulus of NFs. Another reason behind the greater strength of zein NF with increased volume of Nano-CUR could be the occurrence of an increase in the thickness and uniformity of nanofibers.

Nano-CUR has been observed to be well dispersed in zein solution upon the addition of oil/water Nano-CUR, which is probably due to the good mixture of water and acetic acid. Being in accordance with the SEM and fluorescent microscope images, the appearance of Nano-CURs can not be observed on the surface of nanofibers since they had been pushed towards the core of these substances. Apparently, as the polymer jet begins to move towards the collector, the acetic acid-soluble zein polymer is pulled further outwards due to the higher evaporation rate of acetic acid, while the aqueous phase that contains Nano-CUR shows a tendency to further move inwards. The in vitro release study of zein and zein/Nano-CUR NFs has displayed the occurrence of two phases throughout the releasing process of Nano-CUR from NFs, which had initially involved a burst release phase and a sustained release phase in the following. The burst release of Nano-CUR from the scaffold is caused as they move closer towards the surface of nanofibers. However, the impregnated Nano-CURs that exist deeper inside the polymer scaffold require more time to be released, which results in the creation of plateau phase. According to the obtained results, increasing the in vitro cumulative release and reducing EE% is aligned with the occurrence of an increase in the portion of Nano-CUR, which can be legitimized along with the decreasing diameter of NF. Causing a reduction in the NF diameter can lead to an increase in the NF surface ratio and leave less available space inside the NF for the encapsulation of Nano-CUR; therefore, it can be assumed that a higher release is inevitable throughout the NFs that contain a smaller diameter. In conformity with the observations, there has been a higher amount of burst release in the case of zein/Nano-CUR 15%, when compared to the results of 10% and 5% NFs, which could have been related to the narrower diameter of NFs. It has been reported by previous studies that thinner NFs had been able to display a faster in vitro release due to containing a larger surface area and wider contact with the outside environment^20^. In addition, the encapsulation of Nano-CUR in zein NFs has been more than 90% in our work, which proves the applicability of electrospinning technique for this particular purpose. Considering how the continuous release of a drug can be very beneficial, the case of chemotherapy drugs can be taken as an example since the effective concentration of this drug in the blood is preserved between the lowest effective levels and the maximum tolerable level during long periods^46,77,78^.

Results have indicated that the WVTR of zein NFs had been significantly higher than the scaffold of zein/Nano-CUR 10% and 15%. As it is known, controlling the occurrence of water vapor exchange through the nanofiber scaffolds is necessary for providing the proper repair of wounds, since high WVTR can cause rapid wound dehydration and result in leaving scars^79^. A low WVTR can cause the accumulation of exudates, result in delaying the process of wound healing, and increase the possibility of infection^80^. In regards to the performed evaluation of WVTR for burn wounds, Queen et al. have suggested that wound dressings which contain WVTR in the range of 2000–2500 g.m^-2^ could provide a sufficient amount of moisture at the site of wound and avoid the inducement of dehydration^12^. Therefore, it can be assumed that zein/Nano-CUR 10% and 15% can facilitate a sufficient amount of moisture at the site of wound when being compared to zein and zein/Nano-CUR 5%.

Results have indicated that the water-uptake capacity had been significantly increased by enlarging the volume of Nano-CUR, which could have been caused by the hydrophilicity of Nano-CUR. The essential role of water uptake capacity for absorbing extra exudates is quite evident since it can increase the risk of infection and maceration^79^. A high water absorption capacity can effectively increase the absorption of exudates from the wound bed and improve the nutritional perfusion, while water retention is useful in maintaining the moisture of wound bed, which results in increasing the migration of epidermal cells and re-epithelialization^81^. The observed increase in the WVTR and water uptake percentage could have been either caused by the extended hydrophilicity of zein nanofibers, which had occurred after the addition of Nano-CUR (Tween 80 is a hydrophilic nonionic surfactant)^82,83^, or the higher porosity of zein at higher Nano-CUR percentages.

Various studies have investigated the effects of different types of nanofibers on the healing process of skin wounds and according to their date, a better cell attachment and proliferation can be provided by the application of nanofibers that had been synthesized through the electrospinning technique due to containing a high surface-to-volume ratio. The results of these investigations have claimed that nanofibers in the range of 50–500 nm are similar in many aspects to the extracellular matrix and consequently, they can create an optimal environment for both fibroblasts' proliferation and matrix deposition^84^. In order to perform the biological evaluation of synthesized Nano-CUR, we have carried out the in vitro cytotoxicity and intracellular stress oxidative assessments. The obtained results have indicated that next to the lack of causing any cytotoxicity effect on HDF and L929 cell lines, zein/Nano-CUR 5% and 10% NFs have also increased the status of proliferation. According to a comparison between the cytotoxicity and stress oxidative results, the proliferation effect of Nano-CUR can be related to the inducement of a decrease in the oxidative stress. Compared to the case of zein/Nano-CUR 15% nanofibers, the increase in proliferation of zein/Nano-CUR 5 and 10% nanofibers may be the resultant of the existing difference in the release rate and volume of Nano-CUR, which is probably more compatible with the proliferation and removal of free radicals in zein/Nano-CUR 5 and 10% nanofibers. It can be also suggested through this observation that the chemical structure of Nano-CUR in zein NFs is retained after the electrospinning process, while exhibiting cell proliferation and antioxidant properties in the presence of fibroblast cells. Our previous study on the antioxidant activity of emulsion components, oily phase, surfactants, and Nano-CUR has indicated that the antioxidant activity had been related to Nano-CUR. In addition, the effect of Nano-CUR cytotoxicity has not exhibited the inducement of any significant toxicity on Neuro2A cells up to the dosage of around 16 µg/ml^49^.

The antibacterial effect of nanofibers has not displayed any growth of inhibition zone throughout the cases of Escherichia coli, Pseudomonas aeruginosa, and Staphylococcus aureus. There are several studies that have reported the probable antibacterial effects of curcumin and its nano structures^85–88^, however, further investigations are required to figure out why there has not been any detected antibacterial impacts from the zein/Nano-CUR NFs in this study. One of the possible reasons that should be evaluated is the low amount of available Nano-CUR in nanofibers, which can be tested by increasing its content, while another assumption that may be effective is our selected method for investigating the antibacterial effect. Throughout the agar diffusion method, Nano-CURs are limited in being released from all the involved surfaces of nanofibers in order to come into contact with bacteria, which can be considered as one of the disadvantages of this procedure. This factor can be evaluated through the utilization of antimicrobial gradient or dilution methods^89^ since they can provide a better release of Nano-CURs and have them entirely encountered with bacteria.

## Conclusion

In this study, we have prepared zein containing Nano-CUR NFs at three volumes of 5%, 10%, and 15% (v/v) through the employment of electrospinning technique. Thereafter, the physicochemical properties of synthesized NFs, including viscosity, FESEM, FTIR, tensile strength, pore size, encapsulation efficiency, release profile, WVTR, and water-uptake capacity, have been investigated along with evaluating the biological properties such as cytotoxicity and antioxidant assessments on human fibroblast (HDF) and mouse (L929) cell lines. Our work has resulted in obtaining suitable electrospinning parameters that led to the fabrication of uniformed and non-beaded nanofibers. Moreover, we have observed a significant reduction in the mean diameter of zein NFs, as well as the occurrence of an increase in the mechanical properties, by extending the percentage of Nano-CUR. The higher percentage of Nano-CUR loaded NFs have also exhibited higher in vitro release and lower encapsulation efficiency than the other groups of Nano-CUR loaded NFs. According to the results of viability and antioxidant studies, zein/Nano-CUR 10% NFs are capable of providing the best conditions for cell proliferation. Overall, we have suggested in this study that Nano-CUR can be successfully woven in zein NFs and maintain their biological properties.

## Material and methods

### Materials

The following materials have been purchased in analytical grade and used without further purification: Zein (Sigma-Aldrich, Spain), Curcumin (> 99%, Merck, Germany; MW = 368.38 g/mol), Tween 80 (C_64_H_124_O_26_, Merck, Germany), Oil extract of black pepper (Golsorkh-Exir Co., Iran), Glacial acetic acid (> 99.7%, Dr. Mojallali, Iran), Dimethylthiazol-diphenyl tetrazolium bromide (MTT) (> 98%, Sigma-Aldrich, USA), Dichloro-dihydro-fluorescein diacetate (DCFH-DA) (> 98%, Sigma-Aldrich, USA), Dimethyl-sulfoxide (DMSO) (> 99.9%, Sigma- Aldrich, USA), Dulbecco's Modified Eagle Medium (DMEM) (Invitrogen Co, USA), and Ethanol (> 96%, Dr. Mojallali, Iran). Deionized water (DW) has been applied in all of the involved experiments. L929 cell line (NCBI Code: C161) was purchased from Pasteur Institute of Iran and HDF cell line was isolated from human neonatal foreskin samples by Keira et al. protocol^90^. The protocol approved by Ethics Committee of Mashhad University of Medical Sciences (code number: 940891), through obtaining an informed consent from neonatal parents and all the investigations were conducted in accordance with the Declaration of Helsinki.

### Nano-CUR synthesize

The required Nano-CUR has been prepared in accordance with the previously reported method^49^. Briefly, the preparation of Nano-CUR through the means of oil in water (O/W) emulsion has been carried out under three phases. To prepare the oily phase, the mixture of oil black pepper (BP) and surfactant (Tween 80) (1 g : 9 g (w/w)) has been stirred for 15 min and then, 100 mg of curcumin has been stirred in the oily phase while being under magnetic stirring at 500 rpm for 2 h at the specified temperature. The resulting mixture has been homogenized in an ultrasonic bath (DT510, Bandelin, 35 kHz, Germany) for 1 h to remove the existing air bubbles and obtain a homogeneous yellow solution. The final emulsion had been achieved by appending the DW to oily phase (5:1 v/v) and have it stirred at 500 rpm for another 30 min, which had been determined to be 1000 µg/ml at the end of procedure.

### Preparation of zein and zein/Nano-CUR solution

Zein (25% w/w) has been dissolved in acid acetic glacial and stirred for the duration of 12 h to homogenize the solution. To prepare the zein/Nano-CUR solution, 5%, 10%, and 15% (v/v) of the synthesized Nano-CUR has been added into the zein solution to retain the final concentration of 25%. Thereafter, the mixture has been stirred for 12 h through the usage of magnetic stirrer. The prepared zein and zein/Nano-CUR solutions have been left aside for a day at room temperature until the bubbles had been removed.

### Polymer viscosity measurement

The dynamic viscosity of zein and zein/Nano-CUR NFs has been measured by the means of rotating viscometer (Brookfield Co., DV-III Ultra, USA) at room temperature, which had involved the usage of spindle type CC25 at a constant shear rate. The viscosity of zein and zein/Nano-CUR solutions has been reported at the shear rate of 100 s^-1^.

### Electrospinning process

**Figure 8:**
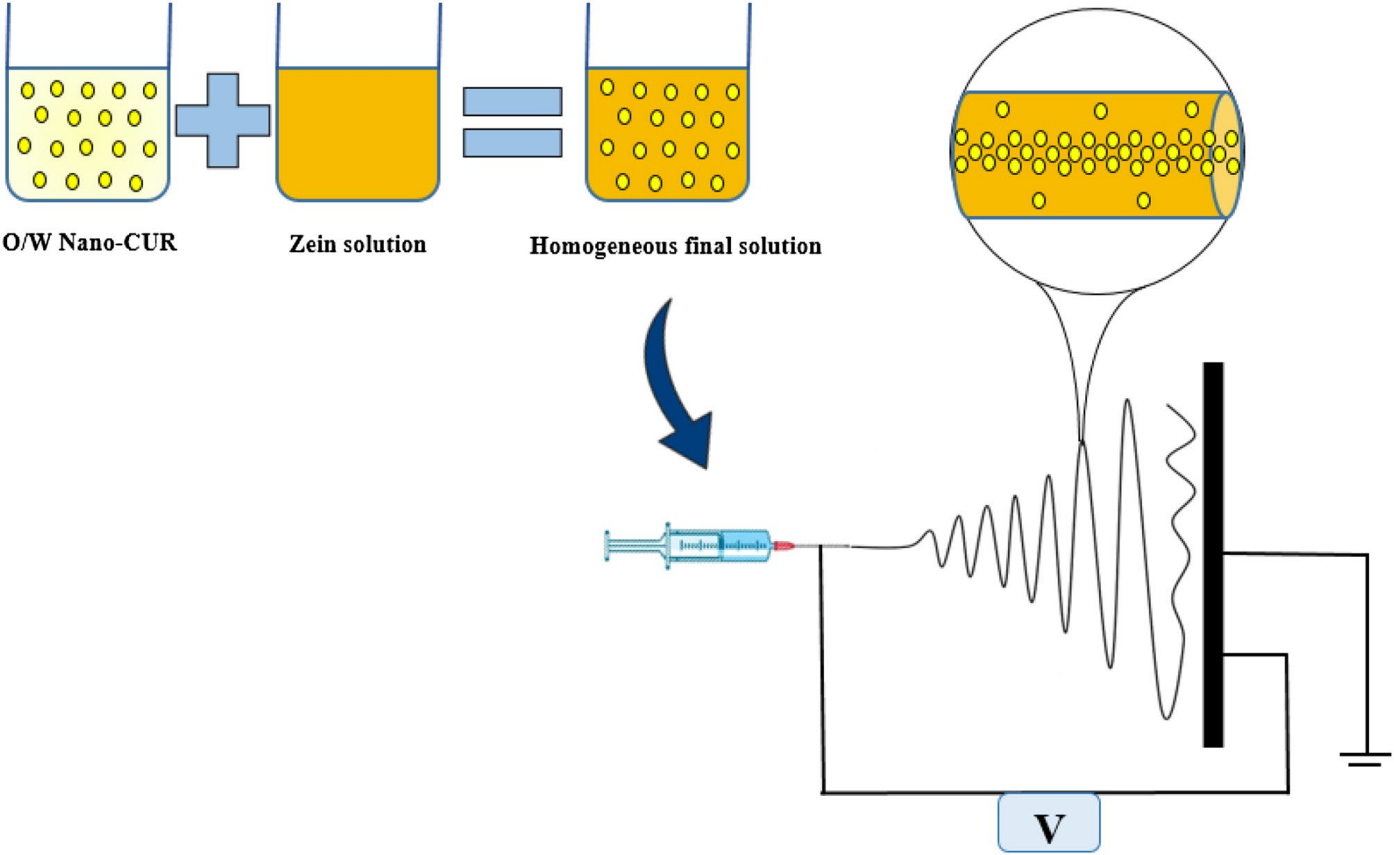
Schematic illustration of the synthesis of zein/Nano-CUR nanofibers.

As it is presented in Fig. 8, Zein and zein/Nano-CUR NFs have been fabricated through the utilization of an electrospinning device that had been equipped with a high voltage power supplier (0–30 kV) (Fnm Co., ES1000, Iran). The available 5 ml plastic syringes have been filled with the solutions of zein and zein/Nano-CUR while being connected to a 16-gauge stainless steel needle. The solutions have been fed into the needle by a syringe pump at 2.5 ml/h, which had been attached to the positive electrode while applying the electrospinning voltage of 25 kV. The created positively charged polymer at the Taylor cone has been observed to move from the tip of needle towards the collector surface that had been covered by aluminum foil. The traveled distance between the needle and collector has been measured to be 150 mm. It should be noted that entire procedure of electrospinning has been carried out at room temperature.

### Surface morphology

We have analyzed the surface morphology of zein and zein/Nano-CUR NFs through the employment of Field Emission-Scanning Electron Microscopy (FESEM) (MIRA3, TESCAN Co., Czech). The images have been taken at the accelerating voltage of 20 kV subsequent to performing the gold coating process (Q150R ES, Quorum Technologies, UK). We have measured the diameters of NF by the usage of Image-J software (https://imagej.net/Fiji) along with the sample size of 100 NF. In addition, the fibers that had contained fluorescent properties have been identified under the Olympus fluorescent microscope (IX53, Japan), which had been equipped with a digital camera (Olympus DP73, Japan).

### Mechanical properties

The mechanical properties of zein and zein/Nano-CUR NFs mats have been determined through the utilization of a universal testing machine (Hounsfield, England) that had contained 10 N of load capacity and 5 mm/min of tensile speed. The NF mats have been cut into 4 × 1 cm sections and a minimum of five measurements had been performed for each specimen. In addition, we have also measured their thickness by the means of digital caliper, which had been carried out in five randomized areas of NFs.

### Fourier transform-infrared spectroscopy (FT-IR)

We have carried out the Fourier transform-infrared spectroscopy (Thermo Nicolet, AVATAR 370, FTIR) analysis of zein-Nano-CUR NFs for the purpose of studying the existing interaction among constituents. The obtained spectra have been displayed at the wavenumber range of 4000–400 cm^-1^.

### Porosity measurements

The porosity of zein and zein/Nano-CUR NFs has been measured through the employment of a micro-volumetric modification of liquid displacement method that had been developed by Moradi et al.^91^. Briefly, subsequent to the immersion of scaffold in a glass pipette (V1) and after having it removed (V2), the induced changes in hexane surface had been recorded by a digital camera, which had been later on evaluated through the usage of ImageJ software (https://imagej.net/Fiji). The porosity measurements have been performed for fifteen times and the results had been reported as mean ± SD. The pore volume (%) can be calculated through the following equation (Eq. 1):

1 $$ {\text{Total}}\;{\text{pore}}\;{\text{volume}} = \frac{V2}{{V1 + V2}} \, \times \,{1}00 $$

### In vitro drug release study

The in vitro cumulative release study of Nano-CUR from zein NFs has been carried out in a specified immersion period for the duration of 20 days. As the next step, the membranes have been cut into the diameters of 2 × 1 cm^2^, accurately weighed, immersed in 4 ml of phosphate buffer saline (PBS, pH = 7.4), and incubated in the shaker incubator (JTS 40, Iran) at 37 °C. At the given time intervals, the samples have been transferred to a new medium and analyzed by the utilization of UV-spectrophotometry (CE9500, CECIL, UK) at 425 nm. We have performed all of the involved release studies in triplicate and had the obtained results reported as mean ± SD.

### Determination of encapsulation efficiency (%)

Encapsulation efficiency is known as the percentage of a drug that has been successfully entrapped after the formulation process. The encapsulation efficiency percentage of Nano-CUR in NFs has been calculated by analyzing the amount of existing Nano-CUR in the supernatant of 2 × 1 cm^2^, having it accurately weighed, and performing the centrifugation of NFs at 15,000 g for 15 min. Furthermore, the optical absorbance has been measured at 425 and 630 nm (as reference wavelength) by the usage of UV–visible spectrophotometer (CE9500, CECIL, UK), while the amount of Nano-CUR in supernatant has been determined through the standard curve of designated concentrations. Being the remaining amount of Nano-CUR in NF, the encapsulation efficiency (%) can be calculated by applying the following equation (Eq. 2**)**. We have performed all of the EE% studies in triplicate and had the obtained results reported as mean ± SD.

$$EE\left(\%\right)=\frac{EncapsulatedNano-CUR}{TotalNano-CUR}\times 100(2)$$

### Water-uptake capacity

To calculate the absorption capacity of wound exudates by nanofiber scaffolds, the water-uptake capacity has been calculated through the following Eq.^92,93^.

$$Water-uptakecapacity\left(\%\right)=\frac{Ww-Wd}{Wd}\times 100$$

In which W_d_ stands as the primary sample weight (1 × 1 cm) and W_w_ represents the sample weight subsequent to being immersed in PBS for 24 h at room temperature. We have repeated the process of water- uptake capacity for three times in regards to each sample and had the results reported as mean ± standard deviation.

### Water vapor transmission rate (WVTR)

Nanofiber scaffolds have been exposed to an open round container with a diameter of 1.2 cm for the purpose of calculating the amount of Water vapor transmission rate. In the following, we have filled the container with 5 ml of sterilized water, which is about one quarter of the container's height, and had the vessel placed in an oven at 35 °C with the humidity of 35% for 24 h. Lastly, the WVTR has been calculated by the usage of the following Eq.^12,92^.

$$WVTR=\frac{\Delta W}{A}$$

where ΔW represents the change of water weight (g) and A is the open surface area of the container (1.13 × 10^–4^ m^2^). A round container without nanofiber scaffolds has been considered as a control group. The amount of WVTR has been calculated for three times in regards to each sample and the results had been reported as mean ± standard deviation.

### In vitro cytotoxicity study

We have evaluated the cytotoxicity of zein and zein/Nano-CUR NFs by performing the MTT assay on human dermal fibroblast cell line (HDF) and mouse fibroblast cell line (L929)^34,35,94^. The HDF and L929 cells have been cultured in high glucose cell culture medium (DMEM) while containing 10% FBS and 0.5% penicillin/streptomycin, which had been then incubated at 37 °C with 10% CO_2_ and 90% humidity. The UV sterilized, 8 mm-circular NFs have been positioned at the bottom of 96-well plate and pre-wetted with culture medium at 37 °C. Subsequent to reaching a confluency of 70%, the cells have been harvested with 0.25% trypsin and 5000 cells, in passages of 3 or 4, which had been afterwards seeded in each well of the NFs membranes. As the next step, we have incubated the plate at 37 °C with 10% CO_2_ and 90% humidity for allowing the cells to proliferate on the membrane for 48 h. Meanwhile, Non-seeded membranes have been considered as the negative control. Subsequent to 48 h, 10 μl of MTT (5 mg/ml) in PBS has been added to each well and once 4 h of incubation at 37 °C had passed, the wells have been emptied from culture media and replaced with 100 μl of DMSO to dissolve the existing formazan crystals. To conclude the procedure, the optical absorbance has been measured at 540 and 630 nm (as reference wavelength) through the usage of a microplate reader (Epoch, BioTeck, USA). All of the involved experiments have been repeated for five times.

### In vitro Antioxidant activity

The oxidative stress of zein/Nano-CUR NFs has been evaluated by utilizing a DCFH-DA colorimetric quantitative method^95,96^. We have seeded the HDF and L929 cells on the membranes that had been positioned at the bottom of each well, which had been thoroughly described in the section of cytotoxicity method. The prepared 96-well plate has been incubated at 37 °C with 10% of CO_2_ and 90% of humidity, while the non-seeded membranes have been considered as the negative control. Subsequent to 48 h, the supernatant of cells has been evacuated and 10 µL of freshly prepared CFH-DA dye (10 μM) had been added to the FBS free medium. Once 40 min of incubation at 37 °C had passed, the fluorescence intensity has been recorded by the employment of a fluorescence plate reader (VICTOR X5, PerkinElmer) in the excitation of 485 nm and the emission of 630 nm. We have repeated every experiment for five times.

### Agar diffusion antibacterial test

Three bacterial strains of Escherichia coli, Pseudomonas aeruginosa (Gram-negative), and Staphylococcus aureus (Gram-positive), which are known as the most common hospital skin wound bacteria, have been exerted to evaluate the antibacterial properties of nanofiber scaffolds through the means of agar diffusion method. In this regard, we have carefully spread 100 µl of bacterial solution (10^6^ CFU/ ml) on the mueller hinton agar plate and had zein and zein/Nano-CUR NFs (1 cm^2^) placed on the plate surface. Vancomycin (30 μg), gentamicin (10 μg), and ciprofloxacin (10 μg) antibiotic discs have been applied as the positive control for Staphylococcus aureus, Escherichia coli, and Pseudomonas aeruginosa, respectively. The inhibition zone has been examined after 24 h of incubation at 37° C.

### Statistical analysis

Statistical analysis was evaluted by two-way analysis of variance (ANOVA) and Tukey post-hoc test. A p-value 0.05 was considered statistically significant. GraphPad Prism 6 software was applied to analyze the data.
